# Can resilience promote calling among Chinese nurses in intensive care units during the COVID-19 pandemic? The mediating role of thriving at work and moderating role of ethical leadership

**DOI:** 10.3389/fpsyg.2022.847536

**Published:** 2022-09-07

**Authors:** Tao Sun, Shu-e Zhang, Hong-yan Yin, Qing-lin Li, Ye Li, Li Li, Yu-fang Gao, Xian-hong Huang, Bei Liu

**Affiliations:** ^1^Department of Health Policy and Management, School of Public Health, Hangzhou Normal University, Hangzhou, China; ^2^Department of Health Management, School of Health Management, Harbin Medical University, Harbin, China; ^3^Department of Humanities and Social Sciences, Harbin Medical University, Daqing, China; ^4^Department of Administration, School of Law, Zhejiang University City College, Hangzhou, China; ^5^Institute of Hospital Management, Qingdao University, Qingdao, China; ^6^Department of Laboratorial Science and Technology and Vaccine Research Center, School of Public Health, Peking University, Beijing, China

**Keywords:** ICU nurse, resilience, calling, thriving at work, ethical leadership, COVID-19 pandemic

## Abstract

**Background:**

Nurses working in the intensive care unit (ICU) clung tenaciously to their job during the COVID-19 pandemic in spite of enduring stressed psychological and physical effects as a result of providing nursing care for the infected patients, which indicates that they possessed a high degree of professionalism and career calling. The aim of this study was to explain the associations between resilience, thriving at work, and ethical leadership influencing the calling of ICU nurses.

**Methods:**

From December 2020 to January 2021 during the COVID-19 pandemic, a cross-sectional survey of 15 provinces in China was conducted using an online questionnaire. A total of 340 ICU nurses (effective response rate: 64.89%) completed sufficient responses to be used in the study. Sociodemographic factors, job demographic factors, resilience, calling, thriving at work, and ethical leadership were assessed using the questionnaire. General linear modeling (GLM), hierarchical linear regression (HLR) analysis, and generalized additive model (GAM) were performed to examine all the considered research hypotheses.

**Results:**

Resilience was positively and significantly associated with calling. Moreover, thriving at work partially mediated the relationship between resilience and calling. The indirect effect of resilience on calling was 0.204 (*p* < 0.0001), and the direct effect of resilience on calling through thriving at work was 0.215 (*p* < 0.0001). The total effect of resilience on calling was 0.419 (*p* < 0.0001). In addition, ethical leadership played a moderating role in the relationship between resilience and calling (β = 0.16, *p* < 0.05).

**Conclusion:**

Greater resilience can positively predict increased calling among Chinese ICU nurses during the COVID-19 pandemic. Moreover, thriving at work is a mechanism that partly transmits the positive effects of resilience on calling. Overall, nurses possessing greater resilience tend to maintain thriving at work in the face of such adversity, further resulting in subsequently increased calling. Besides, findings suggest that there is stronger influence of resilience on calling among nurses working in an organization managed by an ethical leader. The current findings may offer two insights for nursing practitioners and policymakers in the postpandemic world. First, resilience training and intervention are necessary to foster nurses' sense of thriving at work in the nursing industry, further promoting career calling. Second, better training and effort on the development of ethical leadership for leaders in nursing practice are essential to encourage followers to engage in social learning of ethical behaviors and abiding by normatively appropriate conduct, further enacting prosocial values and expressing moral emotions.

## Introduction

The COVID-19 pandemic has dramatically affected medical institutions and professionals, leading to unprecedented pressure, which is challenging for the healthcare workforce, especially the nurses (Daly, 2020). COVID-19 placed nurses in the intensive care unit (ICU) at the forefront of battling this pandemic. ICU nurses were enduring stressed psychological and physical effects as a result of providing nursing care for the infected patients in a challenging context (Gordon et al., 2021). Despite suffering from a huge of stress, ICU nurses adhered to strong commitment to care, indicating that they possess a high degree of professionalism and career calling (Sperling, 2021). Even under the circumstances of the pandemic, nurses persisted on own occupational values and still appreciate worthwhile accomplishments, importance of professional challenge, diversity and interest in the job, personal growth and development, and independence in their practice, hinting that existing a calling inspired nurses to devote themselves to battling the COVID-19 pandemic. Indeed, living a calling had a positive effect on frontline nurses' performance through the clinical and relational care they provided (Zhou and Asante, 2021), and its meaning and value were manifested during the COVID-19 pandemic. Furthermore, facing crisis situations, urgent external needs easily lead to trigger nurses' career calling due to spontaneous motivational responses of humans (Zhu and Chen, 2021). The experience of a calling can lead a person to feel called to do something that is meaningful, satisfying, and worthwhile, thereby contributing to the construction of a positive self-identity (Dalla Rosa et al., 2019), further causing a series of positive consequences for individuals and organizations. In fact, many nurses have presented with a high degree of career calling and positive engagement in defending the pandemic for the past 2 years. Moreover, there are studies about the correlates and consequences of calling in the field of organizational psychology, but relatively less about its antecedents (Lysova et al., 2019) and the conditions and mechanisms explaining why antecedents have positive and/or negative effects on individual calling (Lysova et al., 2019), especially in emergency situations. In general, there are several gaps in understanding what drives nurses' career calling. First, despite these obvious antecedent variables of living a calling, the relationship between individual psychological resilience and calling lacks evidence. Second, we know less about the mediating mechanisms of the relationship between resilience and calling, leaving it unknown why individual psychological resilience positively affects nurses' career calling during a challenging context. Third, there is a paucity of research on boundary conditions of the relationship between resilience and calling, especially in the nurse group. For sure, less research has paid attention to concerning ICU nurses' calling, and its association with resilience, especially their mechanisms and boundary conditions. Empirical research is needed to explain what antecedents can influence nurses' career calling and what variables can play the mediating and moderating role in relations between them.

Resilience is an individual enduring trait or characteristic that can protect the wellbeing and mental health despite exposure to physical and/or psychological burden. The tailored, discipline-specific and sustainable resilience training for healthcare professionals has been widely carried out in the field of theory and practice. Nurses work under constant stressful situations, which place them at increased risk of developing mental disorders, making resilience an essential quality of their personality to manage occupational stress and avoid job burnout. A resilient nurse potentially has positive coping strategies and psychological resources at the time of unprecedented pressure, suggesting that resilience is likely to enhancing the level of career calling. Understanding the relationship between resilience and calling and their mechanisms and boundary conditions may provide an insight into how they present interaction effects in a highly stressed environment.

Thriving as the psychological state presents individuals with experience both as a sense of vitality and learning. People who are thriving experience growth and momentum marked by both a sense of feeling energized and alive (vitality) and a sense that they are continually improving and getting better at what they do (learning) (Porath et al., 2012). Thriving nurses are less susceptible to burnout and more likely to exhibit an active role in developing a successful career calling, which is particularly important given a challenging context caused by the COVID-19 pandemic. Moreover, given the high degree of self-regulation capacity facing stressful situations, a resilient nurse is more likely to have a positive thriving experience, which potentially contributes to enhancing the level of career calling. Therefore, we speculate that thriving at work mediates the relationship between resilience and calling.

Ethical leaders are characterized as honest, caring, and principled individuals who used to make fair and balanced decisions. Given that supervisors can do so by demonstrating honesty, integrity, altruism, and other such traits through their behaviors and actions (Anser et al., 2021), ethical leaders are more likely to reinforce or enlarge the positive effect of psychological attributes of subordinates. As the moral nurse manager, the ethical leader ensures the implementation of ethical standards through punishment and reward system in the nursing team, holds nurses accountable, and makes appropriate decisions to guard the interests and rights of various stakeholders, such as nurses, clinical organization, and society (Abdullah et al., 2019). Thus, we speculate that ethical leadership can contribute to a positive boundary condition of the relationship between resilience and calling, meaning that incorporating ethical leadership into the framework linking resilience and calling can enhance our understanding of the leadership-based differential effects of individual enduring trait or characteristic toward occupational attitude.

**Figure 1:**
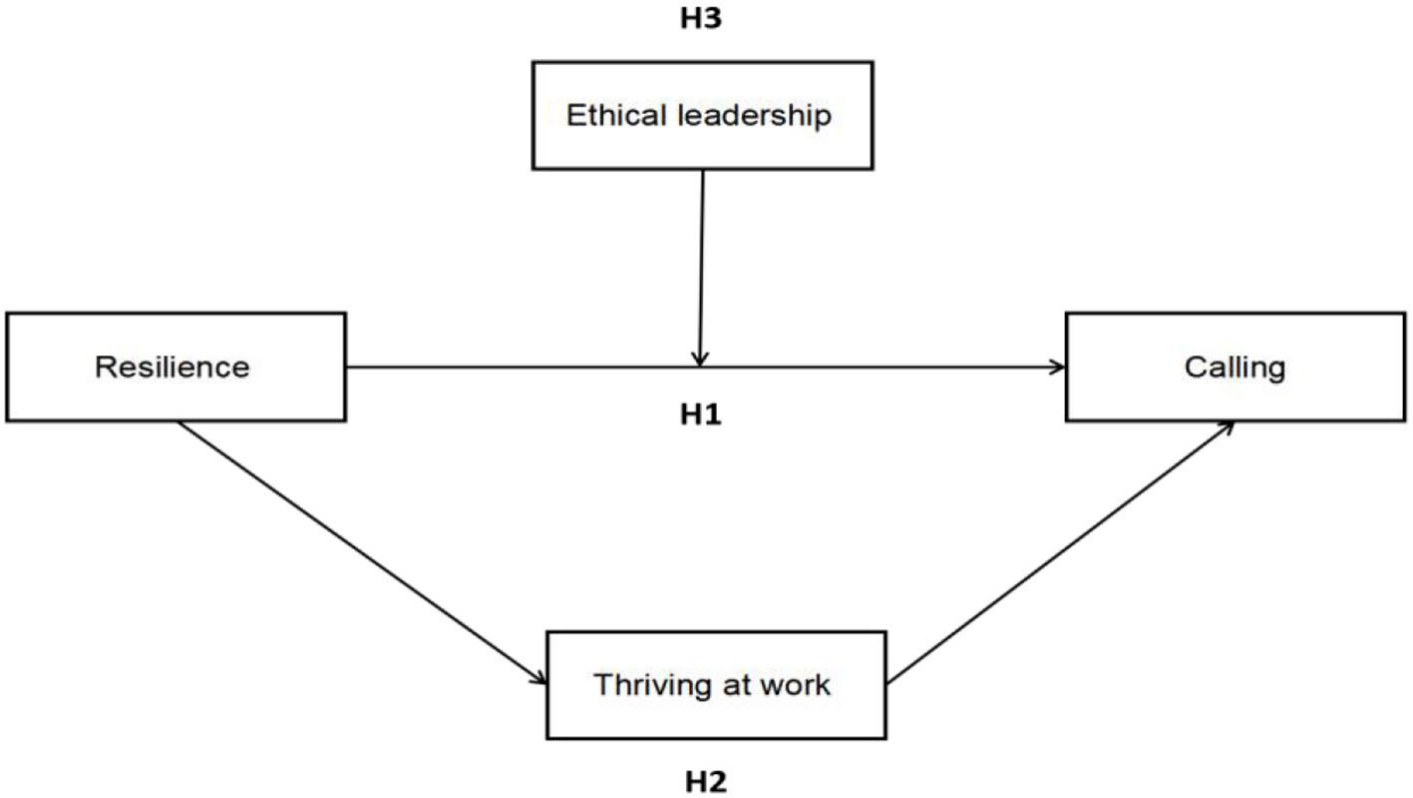
Conceptual model.

Together, this article leans on the conservation of resources (COR) theoretical model that is developed by Stevan Hobfoll to explain resilience and stress in organizational contexts and presents an important explanatory system of individuals' concomitant behavioral responses in stressful situations (Hagger, 2015). In recent years, COR theory has been mostly applied in the research of positive psychology and organizational behavior. COR theory underscores the critical role of resource possession, lack, loss, and gain and depicts personal, which adheres to a basic premise underlying that is what humans are motivated to protect their existing resources and acquire new resources (Hobfoll, 1989). Thus, we proposed an architecture based on the COR theory that aims at explaining how the associations between resilience, thriving at work, and ethical leadership influence the calling of ICU nurse (see Figure 1 for the proposed model). This article is organized as follows. First, a review of the literature on calling and resilience as well as the outline of the hypotheses of the study will be introduced. Second, thriving at work as a mediator, ethical leadership as a moderator, and their relevant hypotheses will be clarified. Third, the main part of the article will be discussed in rest of the article.

## Theory and hypotheses

### Calling

Calling has become an emerging concept for scholars in research on career development and workplace wellbeing as the exponentially increasing volume of published studies on the topic indicates (Lysova et al., 2019). To date, scholars in various disciplines have made various attempts to define calling (Duffy and Dik, 2013). Broadly speaking, a “calling” refers to a person's missions by which she or he is called upon (by a person's own intrinsic motivation, by the social needs, by God, by family legacy, by unknown power, etc.) to engage in a specific occupation or the willingness to serve others (Dik et al., 2012), which means one of the reasons for working, endeavor, and life. Specifically, calling is most often considered as a highly consuming and meaningful career or life that is used to help others, advance the greater good, or influence the society in a certain way, which is driven by a range of sources of the calling, including some internal (e.g., one's own value orientation, interests, skills, fulfillment, and passions), some external (e.g., God, a higher power), and some that may fall in the overlap of internal and external, such as a sense of destiny (e.g., what one is meant to do). Considering different value orientation of work, calling can be divided into three categories, namely, self-oriented and other-oriented nature, and both self- and other-oriented calling (Lysova et al., 2019). Overall, calling consists of three components that—internal meaning/purpose, external summon, and prosocial motivation (Duffy and Dik, 2013)—originating from the bottom of an individual's heart deeply, their sense of meaning and purpose, and their conscientious response to pursue value orientation of a career, respectively (Liu et al., 2019).

Dozens of empirical studies revealed that a sense of career calling has a positive association with the career outcomes and organizational wellbeing (Duffy et al., 2011b). Studies in college students indicated that higher levels of a calling contribute to a series of positive outcomes, such as career self-efficacy (Dik et al., 2008), career hope and decidedness (Zhang et al., 2015), vocational identity achievement (Hirschi and Herrmann, 2012), career preparation behavior (Shin et al., 2018), career adaptability (Kim and Lee, 2016), professional competence (Guo et al., 2014), academic satisfaction (Duffy et al., 2011a), life meaning (Ryan et al., 2011), and reducing dropout (Vianello et al., 2020). Similarly, existing research found that employees living in a calling are prone to exhibit positive organizational outcomes, including organizational commitment (Cao et al., 2019), organization-directed citizenship behavior (Xie et al., 2017), job satisfaction (Xie et al., 2017), work engagement (Xie et al., 2016), psychological empowerment (Kang et al., 2021), psychological wellbeing (Kang et al., 2021), safety performance (Liu et al., 2019), and career success (Xie et al., 2016). Therefore, the value of calling has been confirmed both for individuals and organizations.

### Resilience

Resilience is a hot topic discussed by scholars in various disciplines; it holds similar definitions across the human, social, psychological, and physical sciences (Daly, 2020). To date, although numerous definitions of resilience abound in varied contexts, scholars have not achieved a unified and mutual recognition of an essential understanding of the concept (Sisto et al., 2019). Generally, there are three types of perspectives on defining complex construct of resilience, including a basic attribute (a trait) that is utilized by individuals, a dynamic process (a state) with bidirectional relations to positive and negative factors, and a performance outcome (a function) when challenged by one or more risks (Laird et al., 2019). From the attribute-oriented perspective, resilience is regarded as an individual enduring trait or characteristic that can protect wellbeing and mental health despite exposure to physical and/or psychological burden (Chmitorz et al., 2018). From the perspective of dynamic process-oriented approach, resilience represents a state of positive adaptation and development (Kalisch et al., 2017) within the context of significant adversity, tragedy, trauma, threats, or significant sources of stress (Luthar et al., 2000). As an outcome-oriented understanding, resilience refers to an interactive phenomenon that leads to a successful outcome, demonstrating that some individuals adjust well by showing a stable trajectory with healthy functioning against stress and adversity (Rutter, 2013).

Accumulated evidence supports resilience as a significantly positive factor that supports individual to sustain, regain, or maintain their health, psychological wellbeing, and mental health in the face of challenge (Ungar and Theron, 2020). Literature has documented the importance of a positive outlook in pursuing investments in resilience training and intervention that pay off in the future. Studies found that resilience is negatively associated with neuroticism (Campbell-Sills et al., 2006) and positively related to extraversion (Campbell-Sills et al., 2006) and conscientiousness (Campbell-Sills et al., 2006), and significantly correlates with optimism, self-esteem, social support (Mesarosova et al., 2014), engagement, motivation, and self-compassion (Kotera et al., 2021). Greater resilience contributes to better treatment response in depressed patients (Min et al., 2012), has a positive impact on mental health and functioning (van der Meulen et al., 2020), possesses a protected strength toward post-traumatic stress, depression, and risk for suicidal ideation (Bryan et al., 2015). All in all, existing studies support that resilience is a positive factor in preventing vulnerability, promoting stable functioning, and maintaining positive outcome by adjusting an individual's internal and external resources (Liu et al., 2020).

### Association between resilience and calling

Existing evidence supports that resilience training or intervention is an essentially “positive psychology”—fostering individuals to learn and develop from adversity, trauma, stress, or failure rather than collapse (Mistretta et al., 2018). By coping successfully with traumatic experiences, overcoming the negative effects of risk exposure, or avoiding the negative trajectories associated with risks (Wang et al., 2015), greater resilience not only helps people bounce back from hardship and trauma, but also counterbalances trauma exposures (Fiske et al., 2020), which prevents harm to individual internal meaning, further protecting the calling. A person with high resilience potentially possesses more psychological energy and managing skills (Brown et al., 2020) to enhance his/her professionalism (George et al., 2021) toward own career despite being challenged by various adversities, which can help the individual to maintain career mission, further promoting calling. Moreover, resilience contributes to the sustainable development of competence by learning from failure in the professional fields, which causes increased confidence in the workplace for a person, then preventing the threat of calling (Xu et al., 2021).

In this study, ICU nurses committed themselves to positive reflections on providing nursing care for patients with COVID-19 virus and related their lived experience to the concept of resilience (Thusini, 2020). An experience summarizing from containing the COVID-19 pandemic suggests that nurses should be encouraged to mediate acute stress *via* having a capacity of resilience during the pandemic, which adjusts resources to support ICU nurses to manage stress and promote wellbeing (Thusini, 2020), further maintaining calling. COR theory suggests that resilience is a significant protector under stressful conditions; the latest study confirmed that psychological resilience, which is an individual resource against difficulties, represents a factor that reduces career anxieties of students in the face of pandemic-related negative impacts (Üngüren and Kaçmaz, 2022). A new study found that the engagement in learning, clarity of professional identity, and social support can predict students' calling (Dalla Rosa et al., 2019). Medical professionals combating an invisible enemy are at great personal risk of suffering from financial stressors and burnout during the pandemic. On the contrary, realistic lessons learned from containing the COVID-19 pandemic have indicated that it is needed to equip ICU nurses with positive coping strategies and psychological resources at the time of unprecedented pressure (Thusini, 2020). Previous finding suggests that the more one is searching for his or her meaning in life and intentionally engaging in self-improvement, the more likely she or he is to later experience a calling (Bott and Duffy, 2014). By having intolerance of uncertainty (Tang, 2019), resilience contributes to the engagement in learning from challenging conditions and the sense of achievement from opposing adversity, further increasing the experience of the feeling of success, pride, and meaning, final protecting calling. Thus, we suggested the following hypothesis.

**Hypothesis 1**. Resilience is positively related to calling among intensive care unit nurses.

### Thriving at work as a mediator

Thriving at work refers to a psychological state derived from the joint experience of learning and vitality at work as a critical psychological driver of individual growth and development (Goh et al., 2021), which contributes to individual health and developmental outcomes (Nawaz et al., 2020), indicating that it is an important mechanism for understanding the human dimension of sustainability facing the challenging situations (Nawaz et al., 2020). A meta-analytic review verified that the antecedents of thriving at work consist of unit contextual features, agentic work behaviors, the resources produced at work, and personality traits (Liu et al., 2021). An integrative multilevel review of thriving at work summarizes that social and demographic factors, individual differences, and organizational factors all affect employee's thriving (Goh et al., 2021). Moreover, organizational practices, team characteristics, leadership, and workplace relationships also are the influencing factors of thriving at work (Goh et al., 2021). Being an integrated state by a form of vitality and a sense of learning at work, thriving at work is regarded as a positive psychology, which contributes to both individual and organizational outcomes. At the individual level, thriving at work is positively related to positive health (Walumbwa et al., 2018), lower stress (Um-e-Rubbab et al., 2021), reduced burnout (Hildenbrand et al., 2018), and positive self-leadership (Abid et al., 2021). Moreover, thriving at work has a positive relationship with collective affective commitment (Walumbwa et al., 2018), career growth (Um-e-Rubbab et al., 2021), career adaptability (Jiang, 2017), creative behavior (Alikaj et al., 2021), voice behavior (Sheng and Zhou, 2021), innovative behavior (Wang et al., 2019), life satisfaction (Zhai et al., 2020), and so on. Meanwhile, thriving at work as a mediator has been verified in various fields and career groups. Numerous studies found that thriving at work played a mediating role in a wide range of relations, such as the relation of decent work and voice behavior (Sheng and Zhou, 2021), workplace support and life satisfaction (Zhai et al., 2020), proactive personality and creative behavior (Alikaj et al., 2021), servant leadership and innovative behavior (Wang et al., 2019), positive personality traits and self-leadership (Abid et al., 2021), proactive personality and career adaptability (Jiang, 2017), workplace ostracism and organizational citizenship behaviors toward customers (Han and Hwang, 2021), workplace ostracism and career adaptability (Han and Hwang, 2021), and psychological capital and happiness at work (Basinska and Rozkwitalska, 2020).

Resilience is a dynamic progress of successful adaptability in facing a difficult situation, which contributes to the ability to cope well with adversity and change (McDonald et al., 2016). A previous study indicated that thriving had a positive impact on employability (Hennekam, 2017) and present stress (Um-e-Rubbab et al., 2021), suggesting that it helps individuals to attain success and wellbeing in their careers (Yang and Li, 2021). Thus, a person with a high-level resilience is more likely to have a greater self-efficacy and high sense of employability and post competency through indirect promotion of thriving at work, further contributing to greater calling. In general, according to these reasons, we can conclude that thriving at work tends to mediate the relationship between resilience and calling. Therefore, we suggested the following hypothesis:

**Hypothesis 2**. Thriving at work plays a partial mediator role in the association between resilience and calling among intensive care unit nurses.

### Ethical leadership as a moderator

There are three views on the understanding of ethical leadership. First, ethical leadership is the demonstration of normatively appropriate conduct through personal actions and interpersonal relationships, daily communication, reinforcement, and decision-making (Demirtas and Akdogan, 2015). Second, ethical leadership is regarded as an identity summarized by the comprehensive postures that contain a leader's behaviors, traits, and values, which means that ethical leadership is derived from followers' evaluation after comparing normative expectations (Banks et al., 2021). Third, ethical leadership is a series of signals of leaders, which is assessed by followers during leaders' conduct and performances (Banks et al., 2021). Based on previous theories involving signaling theory, stakeholder theory, attribution theory, social learning theory, social identity theory, social exchange theory, and role congruence theory, the features of ethical leadership could be classified into seven categories as follows: morally appropriate and useful leader behavior, ethical vision creation and sharing, values-based leader behaviors (critique, care, and justice), benevolence-oriented behaviors, and not causing harm (Banks et al., 2021).

A review study suggests that ethical leaders are characterized as honest, caring, and principled individuals who used to make fair and balanced decisions (Brown and Treviño, 2006). For these reasons, when the leader's proactive efforts are to set ethical signals, standards, and use rewards and punishments to see that those standards are followed (Brown and Treviño, 2006), ethical leaders contribute to affecting followers' ethical and unethical behaviors. Thus, ethical leadership tends to produce a series of beneficial outcomes (Dust et al., 2018). At the individual level, numerous studies found that ethical leadership had positively related to reduced job stress and improved performance quality (Schwepker and Dimitriou, 2021), inhibited workplace bullying (Ahmad, 2018), increased professional caregivers' wellbeing and patients' perceptions of quality of care (Gillet et al., 2018), and promoted employee success through raising psychological empowerment and emotional exhaustion. In the organizational dimension, evidence proved that ethical leadership had a positive effective on the organizational outcomes (Khan et al., 2019) and played a critical role in promoting employee loyalty (Fan et al., 2021), rising corporate social responsibility, firm reputation, and firm performance (Nguyen et al., 2021), enhancing leader–member exchange quality and creative process engagement (Nasser et al., 2021), increasing organizational trust and organizational sustainable development (Ilyas et al., 2020), enabling innovation through technology (Lin et al., 2020), influencing employees' perception of the industrial relations climate (Jha and Singh, 2019), determining service employees' engagement in knowledge-hiding behaviors (Abdullah et al., 2019), enhancing exploitative and explorative learning simultaneously (Ali et al., 2022), deducing purchasers' unethical behavior (Ko et al., 2019), and so on.

Ethical leadership can make a favorable external environment for followers through providing ethical signals, standards, and rule of rewards and punishments, which tend to contribute to a positive ethical clime that is beneficial to enhance stronger relationship between resilience and calling. Besides, ethical leadership also is prone to help employee success through promoting psychological empowerment and relieving burnout (Dust et al., 2018). Meanwhile, individual resilience effort contributes to a robust internal system with a positive psychological energy for oneself to optimally “bounce back from” chronic and long-term stressors (Thibodeaux, 2021). To further clarify the sentence,we have been modified the sentence. As you can easily imagine, once emerging interaction between two factors, which is likely to cause the raising sense of career success and loyalty, further arousing internal meaning/purpose, external summon and prosocial motivation those potentially lead to greater calling of staff. It indicats that there is a possibility of existing the interaction effect between ethical leadership and resilience on calling, and suggests that the relationship between resilience and calling is likely to be stronger. Moreover, existing literature also suggests that the moderator role of ethical leadership often be found in many relationship groups (Feng et al., 2019). Thus, we suggested the following hypothesis:

**Hypothesis 3**. Ethical leadership plays a moderating role in the association between resilience and calling among intensive care unit nurses.

Fewer studies provide a clear insight into these complex interrelationships among Chinese ICU nurses. Based on the above review, this study aimed to fill the research gap by analyzing the interrelationships between calling, resilience, thriving at work, and ethical leadership among Chinese ICU nurses using a national survey data and provide scientific evidence. As illustrated in Figure 1, we proposed the following hypotheses: (1) resilience is positively related to calling among ICU nurses; (2) thriving at work plays a partial mediator role in the association between resilience and calling among ICU nurses; and (3) ethical leadership plays a moderating role in the association between resilience and calling among ICU nurses.

## Methods and materials

### Procedure and participants

The study was conducted from 2 December 2020 to 28 January 2021 during the COVID-19 pandemic. Both multistage stratified sampling and convenience sampling methods were used jointly to collect data in this study. In the first sampling stage, Chinese mainland was divided into three regions according to geographic location: eastern region, central region, and western region of China. A total of 15 provinces were selected in this study, containing 6 provinces from eastern region, 4 provinces from central region, and 4 provinces from western region, respectively. In the second sampling stage, considering the convenience of inviting the coordinators, two hospitals were addressed from each sampling province suggested by the coordinators in this study. Approximately 20 ICU nurses of each hospital were invited to complete an anonymous online questionnaire, requiring that the invited nurses need to cover different demographic characteristics using a convenience sampling where coordinators will choose their sample from each hospital based solely on the convenience. Ultimately, 524 nurses participated in this survey. Each selected nurse was invited by the coordinator to click on a webpage link (https://www.wenjuan.com/) to access a self-administered questionnaire. Finally, 340 ICU nurses (effective response rate: 64.89%) provided sufficiently completed responses. For avoiding response bias or non-response bias, we made an immediate interview with a part of non-response nurses by coordinators, realizing that there is no noticeable difference between response sample and either ICU nurses or non-response group, and leading non-response reason was their no immediate response to our invitation.

In total, 524 nurses participated in this survey. Due to incompleteness and false information, 184 questionnaires were excluded, and finally, a total of 340 valid questionnaires were included in the analysis after quality audit and data cleaning, with an effective rate of 64.89%. Among the 340 eligible participants, ~92% were female and 8% were male. A total of 122 (35.88%) participants were from the eastern region, 77 (22.65%) from central region, and 141 (41.47%) from western region of China. A total of 259 (76.18%) had a bachelor's degree; 236 (69.14%) had a junior title; and 229 (67.35%) were married.

### Instruments

#### Measurement of resilience coping (Cronbach's alpha = 0.858)

Brief Resilient Coping Scale (BRCS), developed by Sinclair and Wallston (2004), a four-item measure, was used to assess ICU nurse' resilience coping capacity and skill to capture tendencies to cope with stress in a highly adaptive manner. This instrument also was extensively adopted during the COVID-19 pandemic in the hospital industry (Sehsah et al., 2021). The items have five optional responses format, where 1 = “does not describe me at all,” 2 = “does not describe me,” 3 = “neutral,” 4 = “describes me,” and 5 = “it describes me very well.” The sample item is “I look for creative ways to alter difficult situations.” A higher score reflects a higher skill of resilience coping of nurses.

#### Measurement of calling (Cronbach's alpha = 0.867)

Brief Calling scale (BCS) developed by Dik et al. (2012), was used to measure ICU nurses' career calling. This instrument contains two subdimensions for assessing the presence of, and search for, a calling, including four items. Responses were scored on a five-point Likert scale from 1 (not at all true of me) to 5 (totally true of me). The sample item is “I am searching for my calling as it applies to my career.” A higher score presents a higher career calling of nurses.

#### Measurement of thriving at work (Cronbach's alpha = 0.834)

Thriving at work scale developed by Porath et al. (2012) was used in this study to assess the two subdimensions of nurses' thriving, containing 10 items. Responses were scored on a five-point Likert scale from 1 (strongly disagree) to 5 (strongly agree). The sample item is “I am looking forward to each new day.” Furthermore, according to a previous empirical study on thriving at work, although it has two dimensions, scholars usually calculate the average score for subsequent analysis (Yan et al., 2021). A higher score indicates a higher thriving at work of nurses.

#### Measurement of ethical leadership (Cronbach's alpha = 0.951)

A revised version of ethical leadership scale (Shu and Liang, 2015) developed by an original scale of Brown et al. (2005), containing eight items, was used in this survey. Responses were scored on a five-point Likert scale from 1 (strongly disagree) to 5 (strongly agree). The sample item is “Listens to what employees have to say.” A higher score reflects a higher ethical leadership style for subordinate nurses.

### Statistical analysis

We used SPSS (version 20.0, IBM, Armonk, NY, USA) for data cleaning and Cronbach's α reliability coefficients for each variable. The reliability and validity coefficient of the questionnaire were 0.892 and 0.918, respectively, using ICC formula. The descriptive statistics of all measures were conducted in STATA (version 15.1, Stata Corp LLC, College Station, TX, USA). General linear modeling (GLM) was used to identify the association between resilience scores and calling scores. Hierarchical linear regression (HLR) analysis was performed to examine the relationship between variables and moderating effect. Generalized additive model (GAM) was used to smooth mediator effect on outcome. β-value and their 95% confidence intervals (CIs) were calculated. All *p*-values were two-sided, and *p* < 0.05 was considered statistically significant.

### Quality control

The purpose and significance of this study were clarified on the first page of the questionnaire. We monitored the collecting progress of the survey every day by two authors. After the deadline, we checked the accuracy of the data, completeness of the data, and excluded the questionnaire if (a) not within the range of this study, (b) taking <180 s (which was confirmed as the minimum answering time in the preliminary survey) to answer the questionnaire, or (c) there were logical contradictions between the answers to the questionnaire. The nature of cross-sectional survey exists in a likelihood of common method variance (CMV) phenomenon, meaning that when the data on independent variables (IVs) and dependent variables (DVs) are self-reported by the same respondent, spurious correlations could result because of the common method used to collect the data, which cannot necessarily be attributed to the underlying phenomenon being tested (Gorla et al., 2010). To ensure that common variance method is eligible, we adopted the anonymous survey for protecting anonymity and reducing respondents' speculation for the purpose of measurement and rationalize the questionnaire design (Podsakoff et al., 2003). Moreover, we performed two tests as per Podsakoff's suggestions (Podsakoff et al., 2012). First, an exploratory factor analysis was performed by entering all measurement items of this study; the results indicated that the largest variance explained by an individual factor was 43.82%, showing that neither a single factor nor a general factor accounts for the majority of the covariance in the measures (Podsakoff et al., 2003), ensuring that CMV is not a significant problem. Second, a confirmatory factor analysis also was performed by modeling all measurement items as the indicators of a single factor, and the results show a poor fitness (Williams and Mcgonagle, 2016). CMV is assumed to be substantial if the hypothesized model fits the data (Chang and Zhu, 2007). Overall, the results of both tests indicate that CMV is not a serious threat to our study.

### Ethical approval

The Institutional Review Board of Harbin Medical University approved this study. All subjects participated in the survey voluntarily, and the information in the database was completely deidentified. As the survey was anonymous, it was impossible to obtain informed written consent due to the nature of online survey. In this case, an informed consent form was included at the beginning of the questionnaire to provide a selection for the invited guests. Completing the questionnaire was therefore considered “informed consent” for participation in the survey. Confidentiality was maintained for all information collected in the survey.

## Results

### Correlations among variables

The means, standard deviations, and Pearson correlation coefficients of the variables are presented in Table 1. As is evident from the finding, all variables were significantly and positively correlated with each other. Thriving at work had a positive correlation with resilience (*r* = 0.589, *p* < 0.01) and career calling (*r* = 0.565, *p* < 0.01). Ethical leadership also had a positive correlation with resilience (*r* = 0.429, *p* < 0.01). Additionally, resilience was closely related to career calling (*r* = 0.483, *p* < 0.01).

**Table 1:** Means, standard deviations (SDs), and correlations of continuous variables.

**Variable**	**M**	**SD**	**1**	**2**	**3**	**4**
1. Resilience	3.957	0.620	1.000			
2. Ethical leadership	4.006	0.731	0.429^**^	1.000		
3. Thriving at work	3.900	0.559	0.589^**^	0.610^**^	1.000	
4. Calling	3.865	0.702	0.483^**^	0.481^**^	0.565^**^	1.000

** *p* < 0.01, correlation is significant at the 0.01 level (two-tailed).

### Association between resilience scores and calling scores

The association between resilience scores and calling scores from GLM analysis is reflected in Table 2. Compared to low-resilience score group, high-resilience score group showed a close connection with calling score (β = 0.562, 95% CI 0.424–0.701). After adjustment by sex, age, and marriage, ICU nurses with a higher resilience score had more calling in this survey (β = 0.593, 95% CI 0.454–0.732). Additionally, a high resilience score was connected to calling score after adjusting for other factors affecting calling in a multivariate model (β = 0.582, 95% CI 0.433–0.730). Overall, the resilience variable was positively and significantly associated with career calling (*p* < 0.0001). The *p-*value for trend is < 0.001. Thus, Hypothesis 1 is supported.

**Table 2:** Association between resilience score and calling score in the ICU nurses.

**Resilience**	**Non-adjusted model**		**Model I** ^**a**^		**Model II** ^**b**^	
	**β (95% CI LL, UL)**	***p*-value**	**β (95% CI LL, UL)**	***p-*value**	**β (95% CI LL, UL)**	***p-*value**
Resilience score	0.548 (0.442, 0.654)	<0.0001	0.559 (0.453, 0.664)	<0.0001	0.546 (0.432, 0.659)	<0.0001
**Resilience group**						
Low group^Ref^	0	0	0			
High group	0.562 (0.424,0.701)	<0.0001	0.593 (0.454,0.732)	<0.0001	0.582 (0.433,0.730)	<0.0001
*p-*value for trend	<0.0001					

a Adjusted for sex, age, and marriage.

b Adjusted for sex, age, marriage, hospital level, education, title, position, and employment type.

### Thriving at work as a mediator in the resilience–calling relationship

Hierarchical linear regression analysis was performed to examine the relationship between resilience, calling, and thriving at work after eliminating the effects of demographic variables (sex, age, marriage, hospital level, education, title, position, employment type). This study regarded “resilience” as the independent variable, “thriving at work” as the mediator variable, and “calling” as the dependent variable. GAM was used to smooth mediator effect on outcome. Table 3 shows that thriving at work played a mediating role between resilience and calling. The indirect effect of resilience on calling was found to be 0.204 (*p* < 0.0001), and the direct effect of resilience on calling through thriving at work was found to be 0.215 (*p* < 0.0001), indicating that Hypothesis 2 is supported. The total effect of resilience on calling was 0.419 (*p* < 0.0001).

**Table 3:** Mediation analysis in generalized additive model (GAM).

	**Estimate**	**95% CI**	**95% CI**	***p*-value**
		**lower**	**upper**	
Total effect	0.419	0.340	0.498	<0.0001
Mediation effect	0.204	0.136	0.279	<0.0001
Direct effect	0.215	0.105	0.320	<0.0001
Proportion mediated	0.487	0.317	0.699	<0.0001

Independent variable: resilience.

Dependent variable: calling.

Mediator variable: thriving at work.

Adjusting variables: sex, age, marriage, hospital level, education, title, position, and employment type.

### Ethical leadership as a moderator in the resilience–calling relationship

**Figure 2:**
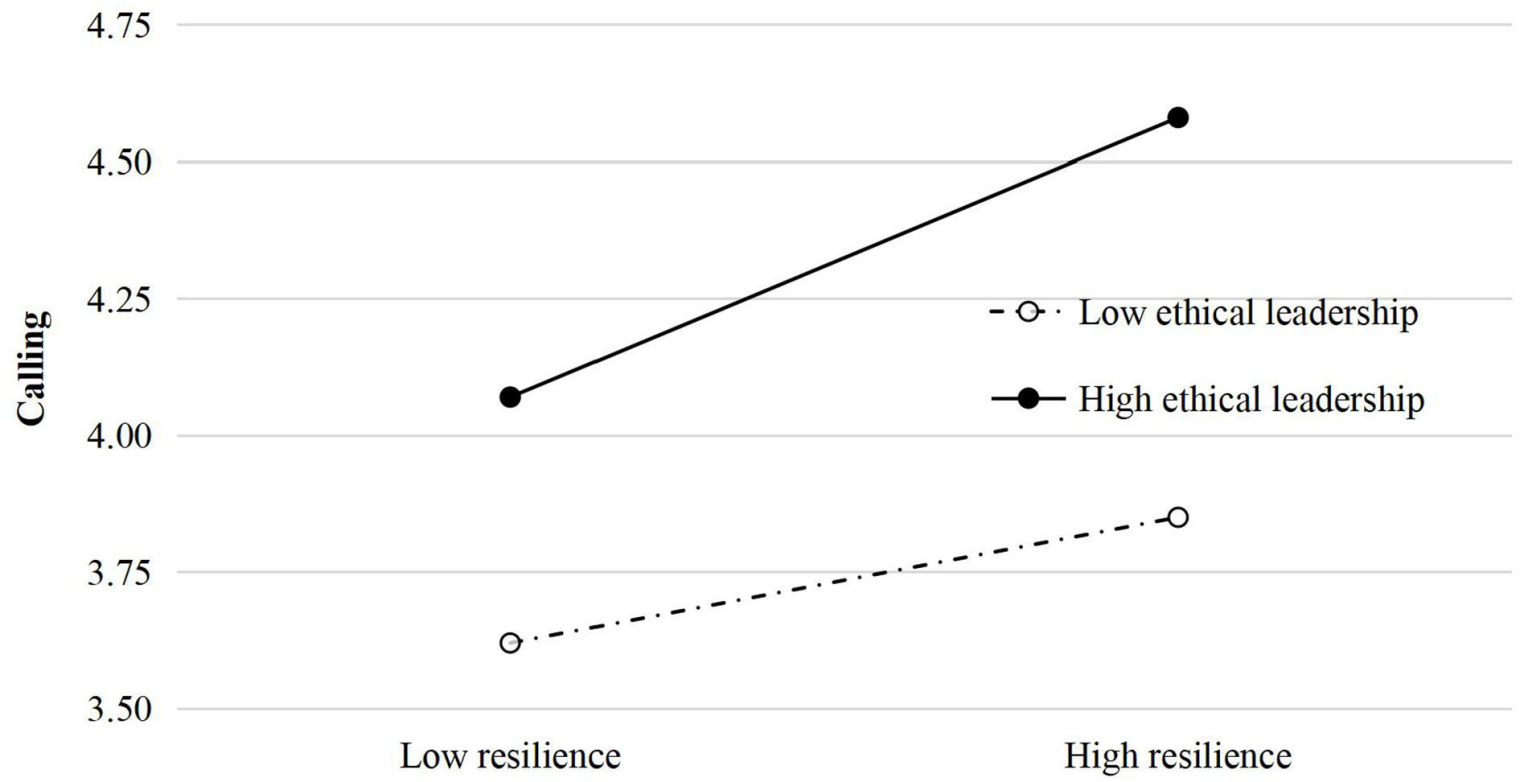
Moderating effect diagram of ethical leadership.

**Figure 3:**
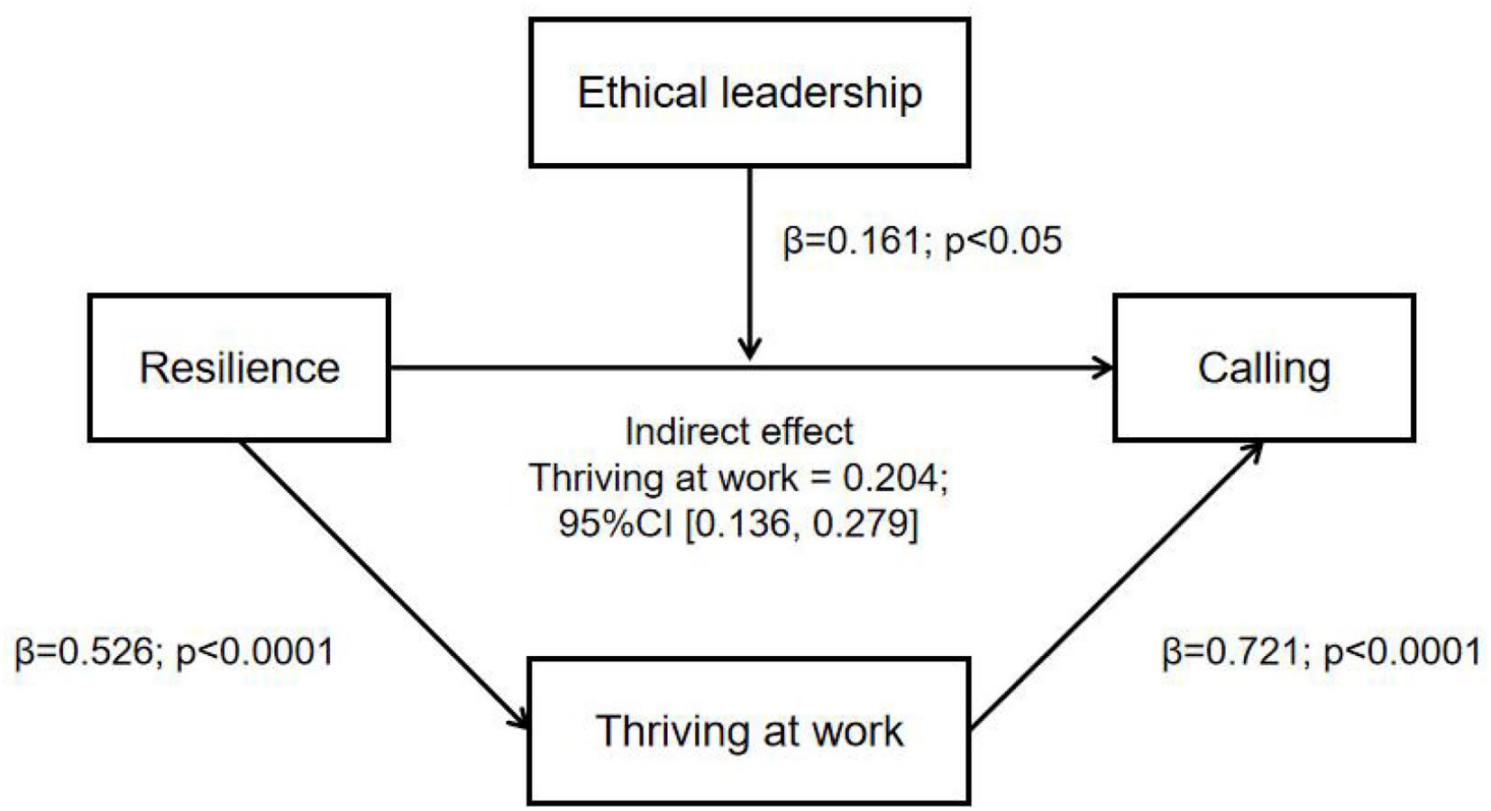
Final model.

Table 4 shows the results of the moderator test for resilience, calling, and ethical leadership in the ICU nurses to provide a breakthrough for the interpretation of the relationship between resilience and calling. As shown in Table 4, the results indicate that ethical leadership (β = 0.16, *p* < 0.05) played a moderating role in the relationship between resilience and calling, thereby suggesting that Hypothesis 3 is also supported. Figure 2 illustrates the differential effects of ethical leadership on the relationship between resilience and calling. Finally, all three hypotheses have been confirmed (see Figure 3).

**Table 4:** Moderated regression analysis.

	**Model I**	**Model II**	**Model III**
	**β (t)**	**β (t)**	**β (t)**
**Constant**	3.87**	3.87**	3.83**
	(115.78)	(123.22)	(113.30)
**Resilience**	0.55**	0.39**	0.38**
	(10.15)	(6.86)	(6.75)
**Ethical leadership**		0.32**	0.32**
		(6.77)	(6.69)
Resilience*ethical leadership			0.16*
			(2.37)
R^2^	0.23	0.33	0.34
adjusted R^2^	0.23	0.32	0.33
F	103.08	81.28	56.81
	*p* = 0.00	*p* = 0.00	*p* = 0.00
ΔR^2^	0.23	0.09	0.01
ΔF	103.08	45.82	5.63
	*p* = 0.00	*p* = 0.00	*p* = 0.018

**p < 0.01; *p < 0.05.

## Discussion

Three hypotheses have been supported by empirical evidence of this study, respectively. The results illustrate that resilience was positively and significantly associated to calling. Furthermore, results emphasize the partial mediating role of thriving at work and the moderating role of ethical leadership in the relationship between resilience and calling. Specific discussions are exhibited as follows.

### Positive influence of resilience on calling among ICU nurses

The present finding confirms that resilience has a positive association with calling of ICU nurses, which is consistent with the previous finding that further suggests that resilience among residents emerged as rooted in their calling to the work of medicine. In fact, the meaning and function of resilience have been widely addressed and discussed among the health professionals. The latest national survey among physicians and US workers found that higher resilience has a significant association with a lower risk of burnout, which was consistent with the outcomes of another latest study. Taken together, higher resilience tends to promote psychological wellbeing (Thusini, 2020), career identity, and success (Yang and Li, 2021), through reducing stress and burnout, final contributing to a positive career calling.

A systematic review regarding nurses' resilience proved that greater resilience was positively related to increased job resources (job satisfaction, coping skills, social support, job retention, self-efficacy, and general wellbeing) and negatively associated with job demands (stress, stress disorder, burnout, workplace bullying, and post-traumatic). This study further suggested that resilience training interventions and can proactively help nurses identify or overcome potential problems, thus cultivating job resources and ultimately promoting personal and professional development. Based on such findings, we can conclude that a resilient nurse is more likely to obtain internal meaning/purpose, external summon, and prosocial motivation (Duffy and Dik, 2013), further promoting their calling toward nursing career. Moreover, a resilient nurse cannot easily exhibit withdrawal behavior after suffering from dilemma, which contributes to solid career calling.

In fact, many studies also focused on the resilience of the health professionals during the COVID-19 pandemic, which suggested that resilience has a significant value in protecting health professionals. More importantly, the facing challenges and stressors of stress caused by the COVID-19 pandemic had become an adversity for Chinese ICU nurses, which potentially contributes to greater threats toward their career calling and professionalism. In this case, a resilient nurse has an advantage in adjusting well by showing a stable trajectory with healthy functioning against stress and adversity (Rutter, 2013), thereby protecting their career calling instead of collapse.

Besides, this finding contributes to insight for management practice, that is, essential measures such as positive organizational or environmental factors, leisure activities, social support, and overcoming previous adversity and interventions should be considered in the healthcare workplace to improve resilience of health professionals and career calling (McKinley et al., 2019).

### Partial mediating role of thriving at work in the resilience–calling relationship among ICU nurses

Result indicates that thriving at work played the partial mediating role in the relationship between resilience and calling. A similar study found that thriving at work mediated the relationship between proactive personality and career adaptability (Jiang, 2017), suggesting that thriving at work is a mechanism that partly transmits the positive effects of personality or psychological traits on career outcome. Hence, our finding also illustrates that thriving at work is a critical mediating mechanism in the resilience–calling relationship. A previous study showed that resilient qualities enable high achievers to thrive and perform at extraordinary levels, explaining that greater resilience contributes to the positive and proactive personality, experience and learning, sense of control, flexibility and adaptability, balance and perspective, and perceived social support (Sarkar and Fletcher, 2014), which further inspire a state of thriving at work, then raising career calling. It is well known that resilience is one of the important dimensions of psychological capital. A recent study found that psychological capital had a positive and significant relationship with strengthening learning (Geremias et al., 2020), indicating that resilience is more likely to be associated with learning capacity that is an important composition for individual thriving at work. Moreover, the latest study suggested that resilience seems to be effective for learners as it deals with the capability to effectively manage difficulties in the past and present time in the learning process (Wang and Liu, 2022). Besides, a resilient nurse tends to sustain the threat of adversity, leading to prevent the loss of psychological resources. Because greater resources can help nurses more likely to accelerate thriving at work, which plays a transmitting mechanism further increasing degree of psychological attachment (Zhang et al., 2021), ultimate resulting in a high degree of career calling.

During the COVID-19 pandemic, ICU nurses put all energies and time into their work, which exhibited a state of thriving at work. Resilient qualities contribute to support ICU nurses to manage stress and promote wellbeing (Thusini, 2020) in facing dilemma and threat circumstances, resulting in maintaining calling. In contrast, Chinese health professionals received high praise from all kinds of media and public due to their unyielding bravery in the battle against the COVID-19 pandemic. In this case, a resilient ICU nurse is more likely to devote to thriving at work due to positive adaptability in the face of the COVID-19 pandemic and disasters, through raising a sense of success and wellbeing in their careers (Yang and Li, 2021), thereby resulting in sustaining career calling.

### Moderating role of ethical leadership in the resilience–calling relationship among ICU nurses

This study also identifies that ethical leadership moderated the relationship between resilience and calling. Specifically, the impact of resilience on calling will be stronger in the nursing team with a high-level ethical leadership than those in a low-level ethical leadership. Existing literature suggests that the moderator role of ethical leadership is found in many group relationships (Feng et al., 2019). However, few studies focused on the moderating role of ethical leadership in the resilience–calling relationship. The findings of this study indicate that resilience as individual qualities and traits tends to exhibit an interaction with ethical leadership. An ethical leader used to present honest, caring, and principled features (Brown and Treviño, 2006) that contribute to the development of organizational culture with ethical climate that tends to be regarded as COR for nurses, thus elevating the influence of resilience on career calling. Thus, the relationship between resilience and career calling is more likely to be stronger in a positive team climate derived from an ethical leader. When a nurse leader makes proactive efforts to exhibit ethical signals, standards, and use rewards and punishments to see that those standards are followed (Brown and Treviño, 2006), ethical nurse leaders can affect nurses' ethical and unethical behavior more likely to be shaped, further promoting the greater influence of resilience on career calling because of the ethical leader's influence. Besides, leadership style is a critical element for fostering organizational climate due to the tendency of cultural values of collectivism in China. Culture has an influence on nurses' understanding and explanation of professional values (Pang et al., 2009). Thus, nurse's organizational behavior will be deeply affected by organizational climate and culture through special leadership style after a long-term interaction with leader subordinates. More importantly, Chinese nurses used to pay much attention to leader's personal integrity (Yi) and human connectedness (Ren) and the lowest in moral discipline (Li) that all are ethical standards belonging to Chinese cultural values (Chang, 2008), in which an ethical climate is more prone to contribute to a positive consequence that a resilient nurse maintains greater calling. Given that fostering ethical climate needs a long time, the behavioral expression of ICU nurses during emergency situation (e.g., COVID-19 period) could be shaped in routine nursing work, which indicates that professional training and intervention regarding fostering ethical leadership for nurse leaders in nursing practice is essential for preventing and handling emergencies in the future.

### Theoretical and practical implications

This study contributes to some of the theoretical and practical implications to promote career calling for nurses, especially in the public health emergencies. Theoretically, this study enhances understanding of the relations among resilience, calling, thriving at work, and ethical leadership. Especially, degree-level resilience is related to increased calling, and thriving at work is a mechanism that partly transmits the positive effects of resilience on calling. Moreover, ethical leadership played a moderating role in the relationship between resilience and calling. Practically, the findings of this study suggest that individual character factor, individual working status, and leadership style are all influential in promoting career calling for nurses. An integrated interventions program should be considered to maintain the high level of career calling for nurses, including psychological resilience intervention, and training of sense of thriving at work and development of ethical leadership. Furthermore, investment in nurses' psychological factor, thriving, and ethical leadership is conducive to the formation of the long-term development of the healthcare organization.

### Limitation

This study contributed to a number of valuable discoveries. Nevertheless, several limitations must be considered. First, data were collected using an online survey through a self-report method, which is likely to produce response bias due to the negative effect or social desirability; therefore, a widely used measuring way should be adopted in the future. Second, the non-random sampling method potentially causes sample bias, which might affect the study results. Third is the cross-sectional nature of the variables; ultimately, these results could not be regarded as describing a causal relationship, suggesting that one important direction for future research involves longitudinal studies. Therefore, these limitations need to be addressed in future research in different countries and different cultures. Besides, we did not intentionally collect special variables about work environment and behavioral changes during the COVID-19 pandemic. This study was conducted only in the context of COVID-19 pandemic, suggesting that a specific factor caused by or related to the COVID-19 pandemic should be considered and included in the survey in future research.

## Conclusion

The results indicated that greater resilience can positively predict increased calling among Chinese ICU nurses during the COVID-19 pandemic. Moreover, thriving at work is a mechanism that partly transmits the positive effects of resilience on calling. Specifically, nurses who possessed greater resilience tended to maintain thriving at work in the face of such adversity, which subsequently increased own calling. Besides, the results also found that ethical leadership played a moderating role in the relationship between resilience and calling, suggesting that there is a stronger influence of resilience on calling among nurses working in an organization under management by an ethical leader. This study contributes to two suggestions through conducting this empirical investigation. First, resilience training and intervention are necessary to foster nurses' sense of thriving at work in the nursing industry, further promoting career calling. Second, better training and effort on the development of ethical leadership for leaders in nursing practice are essential to encourage followers to engage in social learning of ethical behaviors and abiding by normatively appropriate conduct, further enacting prosocial values and expressing moral emotions.

## Data availability statement

The original contributions presented in the study are included in the article/supplementary materials, further inquiries can be directed to the corresponding author.

## Ethics statement

The studies involving human participants were reviewed and approved by the Institutional Review Board (IRB) of Harbin Medical University. The patients/participants provided their written informed consent to participate in this study.

## Author contributions

Conceptualization, methodology, software, and writing—review and editing: TS, S-eZ, and H-yY. Formal analysis: X-hH. Investigation: YL, LL, and Q-lL. Data curation: X-hH and Y-fG. Writing—original draft preparation: TS. Visualization, supervision, and project administration: BL. All authors have read and agreed to the published version of the manuscript.

## Funding

This work was supported by the National Natural Science Fund of China (71774045) to TS and funded by the research projects of Scientific Research Foundation for Scholars of HZNU to TS (2019QDL038).

## Conflict of interest

The authors declare that the research was conducted in the absence of any commercial or financial relationships that could be construed as a potential conflict of interest.

## Publisher's note

All claims expressed in this article are solely those of the authors and do not necessarily represent those of their affiliated organizations, or those of the publisher, the editors and the reviewers. Any product that may be evaluated in this article, or claim that may be made by its manufacturer, is not guaranteed or endorsed by the publisher.
